# Acceptability, Swallowability, Palatability, and Safety of Multiple Film-Coated Mini-Tablets in Children Aged ≥2–<7 Years: Results of an Open-Label Randomised Study

**DOI:** 10.3390/pharmaceutics15020701

**Published:** 2023-02-20

**Authors:** Juliane Münch, Carolin Kloft, Madhi Farhan, Vladislav Fishman, Sining Leng, Hans Martin Bosse, Viviane Klingmann

**Affiliations:** 1 Department of General Pediatrics, Neonatology and Pediatric Cardiology, Medical Faculty, University Hospital Düsseldorf, Heinrich-Heine-University, Moorenstraße 5, 40225 Düsseldorf, Germany; 2 Actelion Pharmaceuticals Ltd., a Janssen Pharmaceutical Company of Johnson & Johnson, 4123 Allschwil, Switzerland; 3 IQVIA RDS, Inc., Durham, NC 27703, USA

**Keywords:** formulation, paediatric, mini-tablet, acceptability, swallowability, palatability, open-label, deglutition, film-coated

## Abstract

This single-centre, open-label, randomised, parallel-group study assessed the acceptability, swallowability, palatability, and safety of film-coated, 3 mm diameter mini-tablets in children aged ≥2–<7 years. In total, 300 participants were randomised (2:2:1:1) to receive a single oral administration of 16 (group A) or 32 (group B) mini-tablets with soft food or 16 (group C) or 32 (group D) mini-tablets with water. Children in each group were stratified by age group (2–<3 years; 3–<4 years; 4–<5 years; 5–<6 years; and 6–<7 years). Groups C and D were pooled for statistical analyses. The rates of acceptability (swallowed ≥80% of the mini-tablets with or without chewing), swallowability (swallowed all mini-tablets without chewing or any leftover), and palatability (positive/neutral responses) were ≥80.0%, ≥42.0%, and ≥82.0%, respectively, across the study groups. No marked differences were observed between groups or across age groups. No adverse events or issues of clinical relevance with deglutition were reported. Mini-tablets taken with soft food or water provide a suitable method for administering medicines to children aged ≥2–<7 years. This study was registered in the German Clinical Trial Register (No. DRKS00024617).

## 1. Introduction

Achieving adequate dosing of medicines in the paediatric population while ensuring safety is challenging [1], as children are often unable to ingest standard-sized solid dose formulations and find many oral medications unpalatable [1,2]. In addition, a drug’s pharmacokinetic profile may be influenced by age and body weight, highlighting a potential need for flexible dosing for paediatric populations [1]. There has been a great effort over the past two decades to develop age-appropriate formulations of medicines for paediatric use [1,2,3,4]. While conventional formulations, such as syrups, are commonplace in paediatric medicine, these formulations can sometimes have disadvantages, such as drug instability, poor palatability, the uncontrolled release of active compounds, inconsistent dosing due to incomplete swallowing, and limited knowledge of which excipients are safe for use in the paediatric population [5,6]. A shift towards small solid dosage forms over liquid formulations has been proposed by the European Medicines Agency (EMA) (in 2014) [4] and the World Health Organization (WHO) (in 2008) [7]. Specially designed mini-tablets could help to overcome these challenges, and they are also suited for the administration of a wide range of doses [1]. Mini-tablets have additional advantages of low costs and greater ease of use (i.e., mini-tablets are simply counted and there is no need to measure volumes or reconstitute) and transport [8]. Furthermore, mini-tablets enable the administration of medicine with soft food, which has been reported to improve the mouthfeel of medicine in patients [2].

An increasing number of studies have demonstrated the potential of mini-tablets as a formulation for paediatric medicine [2]. In a randomised, controlled, cross-over study that compared the acceptability of a single 2 mm placebo mini-tablet (coated and uncoated) with that of 3 mL of syrup in 306 infant or pre-school children (aged 6 months to 5 years), mini-tablets were significantly better than syrup in terms of acceptability (*p* < 0.0001) and swallowability (*p* = 0.002). Additionally, the point estimates for swallowability were higher for the mini-tablets (47–88%) than for syrup (39–73%) across all age groups [9]. More recently, the acceptability and swallowability of a single administration of multiple 2 mm placebo mini-tablets were investigated in two age groups (6–23 months, n = 186; 2–5 years, n = 186) [10]. In this randomised, three-way, cross-over study, the younger age (6–23 months) group received 25 mini-tablets, 100 mini-tablets, and 5 mL of syrup, and the older age (2–5 years) group received 100 mini-tablets, 400 mini-tablets, and 10 mL of syrup. In the younger group of children, for both amounts of mini-tablets, superiority to syrup for acceptability (both *p* < 0.05) and swallowability (both *p* < 0.0001) was demonstrated. In children aged 2–5 years, 400 mini-tablets were non-inferior to syrup concerning acceptability (*p* < 0.0003); however, no superiority was detected, and no non-inferiority was demonstrated for the swallowability of 100 mini-tablets versus syrup [10]. In a study of 100 children aged between 2 and 6 years old, the ability to swallow a single 3 mm uncoated placebo mini-tablet varied with age, with 85% of 5-year-old children being able to swallow the mini-tablets compared to only 46% of 2-year-olds [11].

In a study investigating the acceptability and swallowability of mini-tablets and syrup in children aged 6 months to 5 years old, the administration of mini-tablets was preferred to be taken with a drink over soft food; however, the acceptability of 400 mini-tablets was significantly better when administered with soft food compared with a drink (*p* < 0.003) [10].

As 2 mm diameter mini-tablets have been shown to be effective in children aged 2 days onwards [12], we designed the present study to assess the acceptability, swallowability, and palatability of a single administration of multiple film-coated, 3 mm diameter placebo mini-tablets, taken with soft food or water, in children aged ≥2–<7 years.

## 2. Materials and Methods

### 2.1. Study Design and Participants

This open-label, randomised, parallel-group study (German Clinical Trial Register No. DRKS00024617) was conducted at a single centre in Germany between 29 June (first patient’s first visit) and 8 November 2021 (last patient’s last visit). This study included male and female children aged from ≥2 to <7 years who were in- or out-patients (for any reason) at the University Children’s Hospital Düsseldorf, Germany. Participants were required to be able to follow the study procedures (based on an investigator assessment of medical history and a physical examination, including an oral examination, at screening). Children were excluded from the study if they met any of the following criteria: impairment of swallowing mini-tablets as a consequence of acute or chronic illness or oral deformation; allergies, hypersensitivities, or intolerance to any excipients of the mini-tablets; they had eaten within 1 h of the examination and felt sick afterwards; they had a surgical intervention and were not permitted to drink; participation was not in the best interest of the participant or could confound the study assessments; or they had received any drug that causes nausea, fatigue, or palsy per local prescribing information. Full eligibility criteria are shown in Supplementary Table S1.

**Figure 1:**
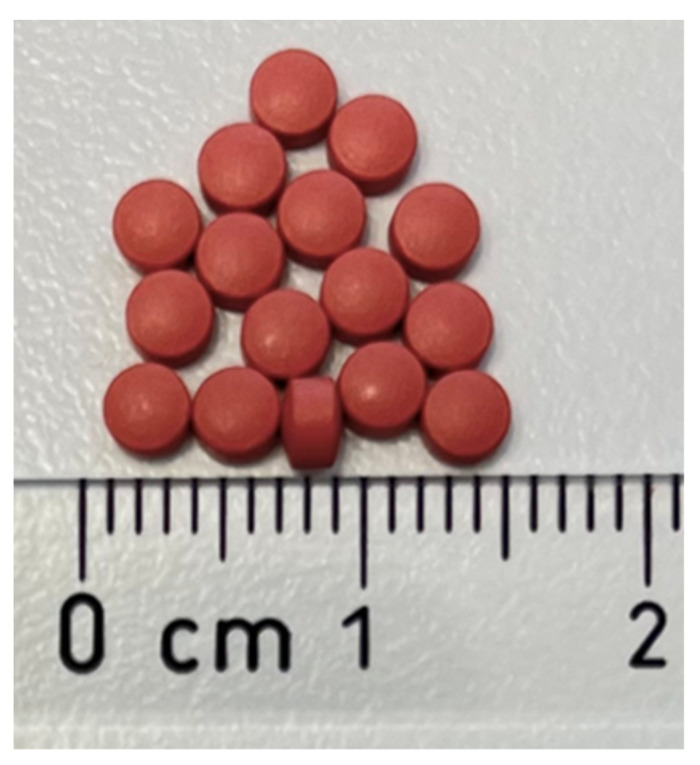
Image of the 3 mm diameter, 2 mm thickness placebo mini-tablets (Excella, Feucht, Germany).

**Figure 2:**
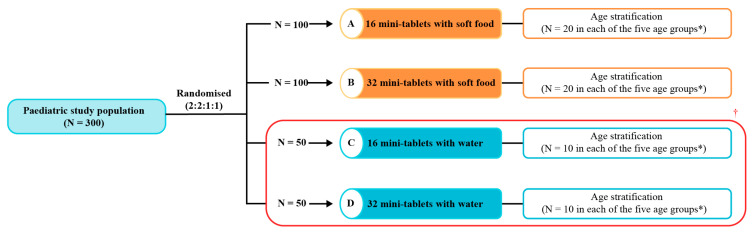
Study design. In total, 300 children were screened, and all of these children were randomised and completed the study. * The age groups were 2–˂3 years, 3–˂4 years, 4–<5 years, 5–<6 years, and 6–<7 years. ^†^ Groups receiving mini-tablets with water (groups C and D) were combined in the statistical analysis. N, number of evaluable subjects.

Study participants who met the eligibility criteria were randomised (2:2:1:1) to receive 16 (group A) or 32 (group B) film-coated, 3 mm diameter placebo mini-tablets (Figure 1) with a teaspoon of soft food or 16 (group C) or 32 mini-tablets (group D) administered with water (Figure 2). The rationale behind assessing soft food versus water was based on the knowledge reported by Klingmann et al. that administration with soft food demonstrated a greater acceptability compared with drinks when assessing the administration of high numbers of mini-tablets in children between 2 and 5 years of age [10]. A permuted block randomisation schedule (computer-generated) was used with stratification by age in each of the four groups: 2–<3 years; 3–<4 years; 4–<5 years; 5–<6 years; and 6–<7 years.

The mini-tablets were administered at the study site under medical supervision. The children were required to be in a fasted state for ≥1 h prior to administration. Mini-tablets were taken orally with soft food (mashed banana, yoghurt, or applesauce) or water (other liquids were not permitted) according to the randomisation scheme. In the soft-food groups, the parents were asked about the child’s preference before choosing the type of soft food. Each child was allowed two attempts at administration, with either a new spoonful of soft food or a mouthful of water for the second attempt. After a first attempt, the spoon and the child’s mouth were inspected (using a tongue depressor and flashlight) to check if there were any left-over mini-tablets and to determine if a second spoonful of soft food or a mouthful of water was required. After a second attempt, if applicable, the child’s mouth was inspected again, and any remaining mini-tablets were counted and documented. The deglutition process and the child’s reactions were thoroughly observed and documented. Treatment-emergent adverse events (AEs) were monitored and recorded on the day of the study.

### 2.2. Mini-Tablets

The 3 mm diameter, 2 mm thickness placebo mini-tablets (Excella, Feucht, Germany) are displayed in Figure 1. The film coating of the mini-tablets (Aquapolish P red 040.67) contained hypromellose, propylene glycol, titanium diocese (E171), iron oxide red (E172), and carnauba wax.

### 2.3. Outcome Measures

The primary endpoint was the acceptability of a single administration of 16 mini-tablets with soft food in children aged ≥2–<7 years (group A). Secondary endpoints were the acceptability of 32 mini-tablets administered with soft food (group B) in comparison to administration with water (groups C and D); the swallowability of 16 and 32 mini-tablets administered with soft food (groups A and B) in comparison to administration with water (groups C and D); and the acceptability, swallowability, and palatability of 16 and 32 mini-tablets administered with soft food (groups A and B) in the age groups. Safety was assessed as a secondary endpoint. Exploratory endpoints included the acceptability, swallowability, and palatability of 16 and 32 mini-tablets administered with soft food (groups A and B) in comparison with water (groups C and D) in the age groups; the number of attempts required for administration by age group; the number of mini-tablets remaining in the oral cavity after administration per age group; and the proportion of children who inhaled or coughed during the ingestion of the mini-tablets.

Pre-defined criteria and definitions for acceptability, swallowability, and palatability are shown in Supplementary Table S2. In brief, acceptability was defined as swallowing ≥80% of the mini-tablets with or without chewing. Swallowability was defined as swallowing all mini-tablets without chewing, with no residuals of the mini-tablets found upon oral inspection. For the assessment of palatability, the immediate reactions of the child after the first administration attempt of the mini-tablets were recorded as pleasant (positive hedonic pattern: tongue protrusion, smack of mouth and lips, finger sucking, and corner elevation), no change (neutral: neutral mouth movements and irregular movements involving lips), or unpleasant (negative hedonic pattern: gape, nose wrinkle, eye squinch, frown, grimace, head shake, and arm flail). A positive or neutral reaction qualified as palatable.

### 2.4. Statistical Analysis

The analysis included all randomised participants who had received mini-tablets.

An acceptability rate of 80% was assumed for the calculation of the sample size. Based on this, a sample size of 100 children per study group was required to obtain sufficient precision (width of the 95% confidence interval (CI) for the estimated rate <10%-points); therefore, groups C and D were pooled for the analyses in order to meet the target sample size of N = 100, aiming to achieve the required statistical precision for the calculation of the acceptability rates of the mini-tablets administered with water.

No formal statistical hypotheses or tests were planned for this study. Demographic characteristics were summarised descriptively. The rates of acceptability, swallowability, and palatability were summarised in contingency tables with frequencies and percentages and were analysed by study group and by age group. Exact 95% CIs were calculated using the Clopper–Pearson method for proportions by study group and using the Miettinen–Nurminen method for between-group comparisons.

The Statistical analyses were conducted using Statistical Analysis System (SAS) version 9.4.

## 3. Results

### 3.1. Participants

A total of 300 children were screened and randomised in the study (group A, N = 100; group B, N = 100; group C, N = 50; and group D, N = 50 (Figure 2)). All 300 children completed the study and were included in the analysis. The median (range) age of the children was 4 (2, 6) years for all study groups. More than half (58.3%) of the children were male, and most children were non-Hispanic or non-Latino (97.3%; Table 1). The demographic characteristics were generally similar across the age groups (Supplementary Table S3), although there was a numerically higher proportion of male participants in the 5–<6- and 6–<7-year-old age groups. Overall, 11 children (3.7%) had abnormal physical examination results at screening; however, these were not considered to be of clinical relevance for conducting the study.

### 3.2. Acceptability, Swallowability, and Palatability of Placebo Mini-Tablets Administered with Soft Food or Water

**Figure 3:**
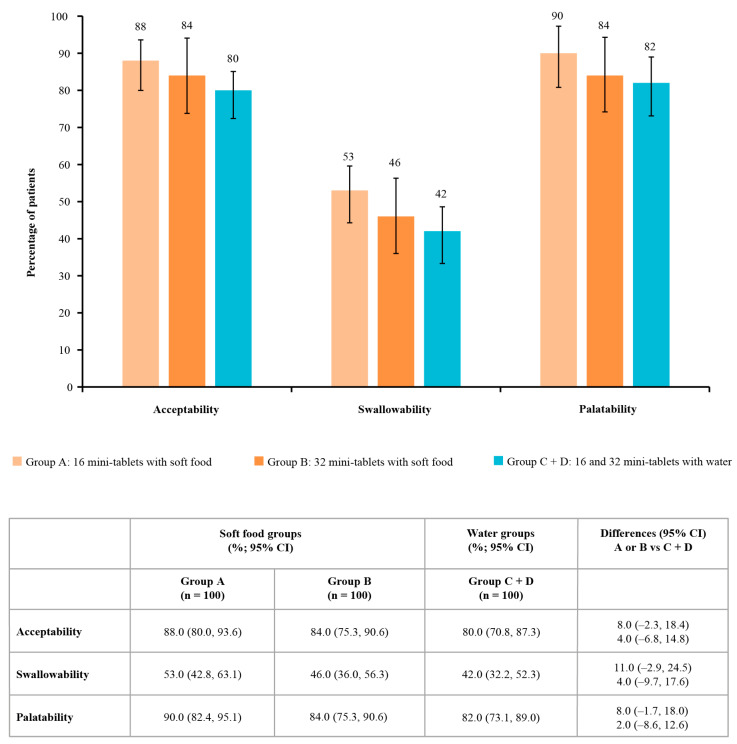
Acceptability, swallowability, and palatability of 3 mm placebo mini-tablets by treatment group. Bars represent 95% CIs. Group A: 16 mini-tablets with soft food. Group B: 32 mini-tablets with soft food. Group C + D: 16 mini-tablets with water and 32 mini-tablets with water, which were pooled for statistical comparisons. Acceptability was defined as swallowing all of the mini-tablets with no chewing or swallowing ≥80% of the mini-tablets with or without chewing. Swallowability was defined as swallowing all of the mini-tablets without chewing, with no residuals of the mini-tablets found in an oral inspection. For the assessment of palatability, the immediate reactions of the child after the administration of the mini-tablets were recorded as positive (pleasant), neutral (no change), or negative (unpleasant) according to the pre-defined criteria. A positive or neutral reaction qualified as palatable. CI, confidence interval; n, number of evaluable subjects.

The acceptability rate for 16 mini-tablets administered with soft food in group A (primary endpoint; Figure 3) was 88.0% (95% CI: 80.0, 93.6). The acceptability was 84.0% (95% CI: 75.3, 90.6) for 32 mini-tablets administered with soft food in group B and 80.0% (95% CI: 70.8, 87.3) for 16 or 32 mini-tablets administered with water in the pooled groups C and D (Figure 3). There were no marked differences (95% CI) in the acceptability rates between groups A or B and the pooled groups C and D (group A vs. C/D comparison: 8.00 (95% CI: −2.3, 18.4); group B vs. C/D comparison: 4.00 [−6.8, 14.8]; Figure 3). In groups C and D individually, the acceptability rates were 82.0% and 78.0%, respectively.

The swallowability rates (i.e., swallowing all mini-tablets without chewing) were numerically higher when mini-tablets were administered with soft food than with water. Swallowability was 53.0% (95% CI: 42.8, 63.1) for 16 mini-tablets administered with soft food in group A and 42.0% (95% CI: 32.2, 52.3) for 16 or 32 mini-tablets administered with water in the pooled groups C and D (Figure 3); there was no significant difference in swallowability between these methods of administration (difference: 11.0 (95% CI: −2.9, 24.5)). The swallowability rates for groups C and D individually were 44.0% and 40.0%, respectively. In children in group B who received 32 mini-tablets administered with soft food, swallowability was 46.0% (95% CI: 36.0, 56.3); the difference compared with the pooled groups C and D was 4.0% (95% CI: −9.7, 17.6; Figure 3), indicating no significant difference.

Palatability was 90.0% (90/100; 95% CI: 82.4, 95.1) for 16 mini-tablets administered with soft food and 84.0% (95% CI: 75.3, 90.6) for 32 mini-tablets administered with soft food (Figure 3); compared with the administration of 16 or 32 mini-tablets with water in 82.0% of children in the pooled groups C and D (95% CI: 73.1, 89.0), these differences were not significant (Figure 3). In groups C and D individually, the palatability rates were 84.0% and 80.0%, respectively.

No AEs or clinically relevant issues during deglutition were reported in any study group.

### 3.3. Acceptability, Swallowability, and Palatability of Placebo Mini-Tablets Administered with Soft Food or Water by Age Group

**Figure 4:**
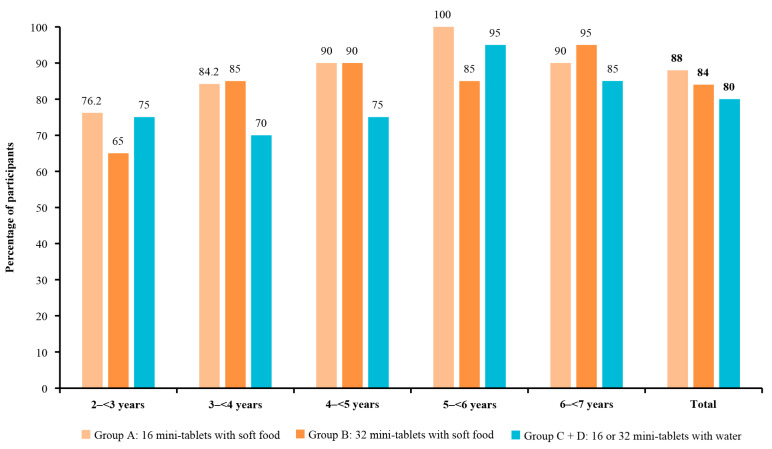
Acceptability of 3 mm placebo mini-tablets by age group.

**Figure 5:**
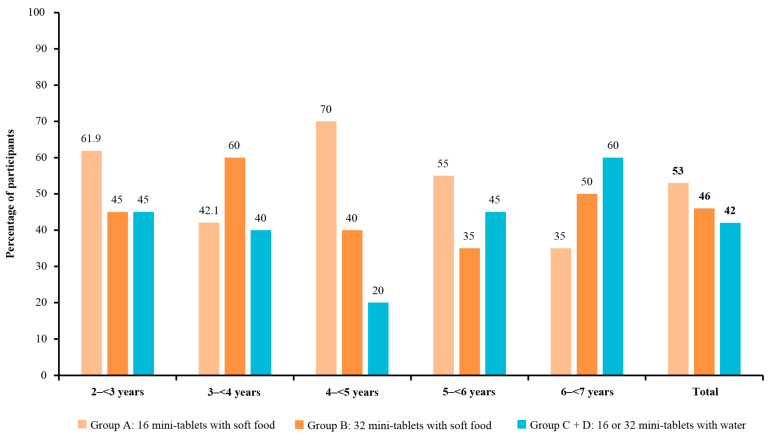
Swallowability of 3 mm placebo mini-tablets by age group. Swallowability was defined as swallowing all of the mini-tablets without chewing, with no residuals of the mini-tablets found in an oral inspection.

**Figure 6:**
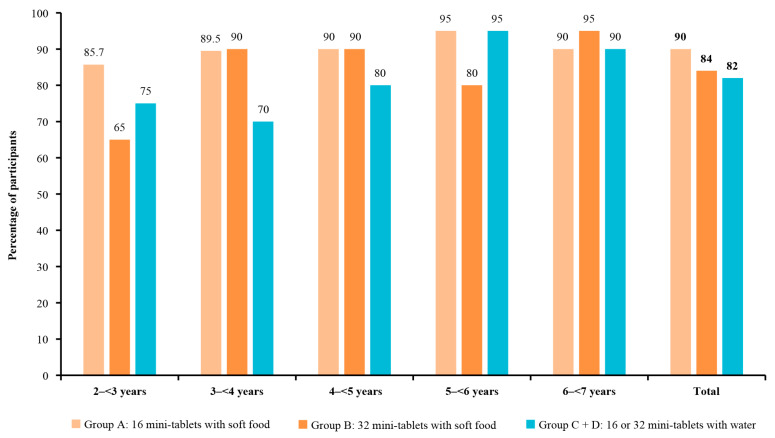
Palatability of 3 mm placebo mini-tablets by age group. For the assessment of palatability, the immediate reactions of the child after the administration of the mini-tablets were recorded as positive (pleasant), neutral (no change), or negative (unpleasant) according to the pre-defined criteria. A positive or neutral reaction qualified as palatable.

The acceptability rates for 16 and 32 mini-tablets administered with soft food generally increased numerically with age, with the lowest acceptability in the youngest age group (2–<3 years) in both study groups (group A: 76.2% (95% CI: 52.8, 91.8); group B: 65.0% (95% CI: 40.8, 84.6)) and the highest acceptability in the group aged 5–<6 years for group A (100.0% (95% CI: 83.2, 100.0)) and the 6–<7 years age group for group B (95.0% (95% CI: 75.1, 99.9); Figure 4 and Supplementary Tables S4 and S5). Across age groups, the swallowability and palatability of 16 and 32 mini-tablets were variable, and there was no trend observed with increasing age (Figure 5 and Figure 6). The children aged 4–<5 years in group A had the highest rate of swallowability of 70.0% (n = 14/20; (95% CI: 45.7, 88.1)), compared with administration of mini-tablets with water (20.0% (n = 4/20)), with a difference of 50.0% (95% CI: 19.4, 71.8) (Supplementary Table S4).

The acceptability, swallowability, and palatability rates for mini-tablets administered with soft food were generally comparable to the rates for mini-tablets administered with water in children of different age groups (Supplementary Tables S4 and S5). The small sample sizes of each age group limited our ability to interpret any numerical differences between the groups.

One child coughed during the ingestion of the mini-tablets (group A), but this was not considered to be of any clinical concern.

Acceptability was defined as swallowing all of the mini-tablets with no chewing or swallowing ≥80% of the mini-tablets with or without chewing.

### 3.4. Exploratory Endpoints

Overall, 11, 15, and 18 children either spat out or refused to take the mini-tablets in groups A, B, and pooled C + D, respectively. Refusal of the mini-tablets was more frequent in the younger age groups than the older age groups.

Among the children who swallowed the mini-tablets without spitting, refusing, or coughing (n = 255), most children (n = 143) had no mini-tablets remaining in the oral cavity after the first attempt of administration with either soft food or water. The successful administration of all mini-tablets on the first attempt was more frequent in groups A (65%; n = 65) and B (54%; n = 54) compared with groups C (34%; n = 17) and D (14%; n = 7).

Of the 114 children who chewed or swallowed some of the mini-tablets (group A, n = 35; group B, n = 39; group C, n = 20; group D, n = 20), the majority of children did not have any leftover mini-tablets in the oral cavity, and this was similar across all groups (group A: n = 34; group B: n = 38; group C: n = 19; group D: n = 19). Only four children had remnants of mini-tablets in their mouths after two attempts of swallowing. The number of mini-tablets that remained in these four cases were 3 (group A), 11 (group B), 4 (group C), and 8 (group D).

## 4. Discussion

This single-centre, open-label, randomised, parallel-group study demonstrated that the acceptability, swallowability, and palatability rates of a single administration of multiple (either 16 or 32) film-coated placebo mini-tablets (3 mm in diameter) with soft food or water in children aged ≥2–<7 years were ≥80%, >40%, and >80%, respectively. Some numerical differences were observed in the exploratory analyses, with higher rates of acceptability, swallowability, and palatability in the groups of children who received mini-tablets with soft food compared with water and higher rates of acceptance in older age groups. However, no significant differences were observed between the study groups or across the age groups for any parameter. No AEs were reported with a single administration of multiple film-coated placebo mini-tablets. These findings suggest that the administration of mini-tablets is a feasible and acceptable approach for the oral administration of medicines in children aged ≥2–7 years.

Acceptability was high for mini-tablets administered with either soft food or water and, as expected, was generally lower in the younger age groups, although 76.2% (group A), 65.0% (group B), and 75.0% (groups C and D) of children aged 2–<3 years accepted the mini-tablets. Generally, the acceptability rates were more favourable in children receiving mini-tablets with soft food compared with water; however, the acceptability rates were marginally better in children aged 2–<3 years receiving 16 or 32 mini-tablets (groups C and D) with water compared with 32 mini-tablets with soft food (group B). These study results also indicate that the film-coated placebo mini-tablets are palatable in children aged ≥2–7 years in terms of mouthfeel and taste.

The swallowability rates were lower (42.0–53.0%) than the rates of acceptability and palatability, reflecting the more stringent criteria for this endpoint, i.e., children were required to swallow all of the administered mini-tablets without chewing. Given that children tend to have a reflex reaction when chewing [10], the observed rates of swallowability are considered acceptable, particularly in the younger age groups (<5 years) who received the mini-tablets with soft food. This single-administration study was not able to assess whether there would be a learning effect (in terms of swallowing in the first attempt rather than chewing) or potentially an increase in the swallowability rate with the repeated ingestion or long-term use of mini-tablets. Importantly, of those 114 children who chewed the mini-tablets, only four had remnants left in their mouths after the second attempt. Therefore, the mini-tablets reached the children’s stomachs in the majority of cases, as demonstrated by the high acceptability rate. This is a key finding showing that mini-tablets could offer a reliable method of administering paediatric medicines. This finding also suggests that the formulation and excipients in these placebo mini-tablets are palatable and well accepted by children. However, the study did not address the potential impact of an active pharmaceutical ingredient on the palatability of these mini-tablets.

There was substantial variation across the age groups in swallowability and palatability. Overall, the findings were promising across the entire age range. The results of a previous study suggested that mini-tablets may only be suitable for children from 4 years old [11]. However, the present results suggest that mini-tablets could be a feasible and safe method of drug administration for children as young as 2 years old and are in line with the findings of other studies, including studies that have demonstrated the acceptance of mini-tablets by very young children, including newborns and infants 6 months to 1 year old [10,12,13,14,15].

The outcomes in the group of children who received 16 mini-tablets administered with soft food (group A) appeared to be slightly better than those in the pooled group who received mini-tablets with water; however, the differences were not significant. The majority of the children in the soft-food groups swallowed all of the mini-tablets in one spoonful, whereas a greater proportion of those in the pooled water groups required two attempts. Our study showed that administering mini-tablets with soft food may be easier and more effective based on these descriptive data; however, this may vary between age groups. For example, the rates of acceptability seemed to be more favourable with water compared with soft food in children aged ≥2–<3 years old.

The strengths of this study included the wide age range of the children that were enrolled. In addition, the study utilised well-defined, validated, and standardised procedures. To the best of our knowledge, this is the first clinical study investigating the acceptability, swallowability, and palatability of multiple coated 3 mm mini-tablets taken with soft food or water. Previous studies have investigated the acceptability of a single uncoated 3 mm mini-tablet [11], a single coated 2 mm mini-tablet [9], multiple uncoated 2 mm mini-tablets [10], and multiple coated 3 mm mini-tablets (administered with jelly) [9,10,11,16].

Several limitations of this study must be acknowledged. The study was conducted in a hospital setting, and the study participants were children who were not seriously ill. Therefore, these results may not be generalisable to severely ill children or chronically ill patients receiving care at home. In addition, the study only investigated a single administration of mini-tablets, and repeated or long-term administrations were not assessed. The administration of the mini-tablets under medical supervision also may not reflect how mini-tablets would be administered to children in a real-world setting. A further limitation is the lack of formal hypothesis testing. The data for the study groups who received 16 and 32 mini-tablets with water were combined, with the aim of providing the statistical precision required for the calculation of the acceptability rates; however, a limitation is that these results for the pooled data may not accurately reflect the acceptability, swallowability, or palatability of the administration of either 16 or 32 mini-tablets administered with water. Although we have reported the rates of acceptability, swallowability, and palatability for groups C and D individually, these were not powered for statistical comparisons with group A or group B. Further investigations of the effects of chewing are also required to determine if the tastes of active pharmaceutical ingredients or excipients, which are normally masked by the film coating, have any impacts on acceptability and palatability, for example, if a bitter active pharmaceutical ingredient would result in lower rates of acceptability and palatability versus placebo. Moreover, it would be interesting to assess whether there is any learning effect with chronic use to reduce chewing and increase the rates of swallowability. Additionally, the viscosity of the soft foods was not analysed; this information would be useful in determining the suitability of each type of soft food that was used to administer mini-tablets in this study. Finally, although safety was assessed in this study, the sample size was too small to draw definitive conclusions on the safety of multiple coated 3 mm mini-tablets administered with soft food or water.

## 5. Conclusions

Film-coated mini-tablets with a diameter of 3 mm provide an appropriate and child-friendly method of administering medicine to children aged ≥2–<7 years when administered with soft food or water. Therefore, these findings may contribute to the shift towards small solid dosage forms (over liquid/syrup), as proposed by EMA [4] and WHO [7].

## Figures and Tables

**Table 1:** Demographic characteristics of study participants.

Characteristic	Group A; 16 Mini-Tablets with Soft Food (N = 100)	Group B; 32 Mini-Tablets with Soft Food (N = 100)	Group C; 16 Mini-Tablets with Water (N = 50)	Group D; 32 Mini-Tablets with Water (N = 50)	Total (N = 300)
**Age, years**					
Mean (SD)	4.0 (1.4)	4.0 (1.4)	4.0 (1.4)	4.0 (1.4)	4.0 (1.4)
Median (range)	4 (2, 6)	4 (2, 6)	4 (2, 6)	4 (2, 6)	4 (2, 6)
**Sex, n (%)**					
Female	48 (48.0)	36 (36.0)	20 (40.0)	21 (42.0)	125 (41.7)
Male	52 (52.0)	64 (64.0)	30 (60.0)	29 (58.0)	175 (58.3)
**Ethnicity, n (%)**					
Hispanic or Latino	5 (5.0)	2 (2.0)	1 (2.0)	0	8 (2.7)
Non-Hispanic or Latino	95 (95.0)	98 (98.0)	49 (98.0)	50 (100.0)	292 (97.3)

N, number of evaluable subjects; SD, standard deviation.

## Data Availability

The data sharing policy of the sponsor is available at https://www.janssen.com/clinical-trials/transparency (accessed on 14 February 2023). As noted on this site, requests for access to the study data can be submitted through the Yale Open Data Access Project site at http://yoda.yale.edu (accessed on 14 February 2023).

## Supplementary Materials

The following supporting information can be downloaded at https://www.mdpi.com/article/10.3390/pharmaceutics15020701/s1, Table S1: Eligibility criteria; Table S2: Definitions and outcomes of acceptability and swallowability; Table S3: Demographic characteristics of study participants by age group; Table S4: Acceptability, swallowability, and palatability of 16 placebo mini-tablets administered with soft food compared with administration with water by age group; Table S5: Acceptability, swallowability, and palatability of 32 placebo mini-tablets administered with soft food compared with administration with water by age group.
