# Supplementary material for: Acceptability, Swallowability, Palatability, and Safety of Multiple Film-Coated Mini-Tablets in Children Aged ≥2–<7 Years: Results of an Open-Label Randomised Study
Source: Pharmaceutics. 2023 Feb 20;15(2):701. doi: 10.3390/pharmaceutics15020701 (PMC9964507; doi:10.3390/pharmaceutics15020701)
Supplement: Supplementary file 1 [file pharmaceutics-15-00701-s001.zip › pharmaceutics-2144827-supplementary.pdf]

Supplementary Table S1. Eligibility criteria

Inclusion criteria

1. Children (males and females) between ≥2–<7 years of age.
2. Based on the assessment of medical history and physical examination (ears, nose, mouth, and throat, respiratory, and gastrointestinal [abdomen]) patients must be able to follow the study procedures.
3. Both parents or their legally acceptable representatives must sign an informed consent indicating that they understand the purpose of, and examination procedures required for, participant obligations as well as risks and benefits of participation in the study, and are willing to allow the child to participate in the study, and likely to comply with examination procedures and restrictions. In case only one parent is present at site to give consent, consent of the second parent may be documented by an independent witness with approval via phone/email/fax. Consent by one parent is acceptable in case of documented sole custody.

Exclusion criteria

1. Any impairment of swallowing mini-tablets as a consequence of:
    a. chronic illness (e.g., cerebral palsy),
    b. acute illness (e.g., sepsis, respiratory distress, gastroenteritis, respiratory tract infection), or
    c. oral deformation (e.g., congenital abnormality, injury, allergy, infection, and surgery).
2. Known allergies, hypersensitivity, or intolerance to any of the excipients of the placebo mini-tablets.
3. Children, who have eaten 1 hour before examination and who afterwards feel sick.
4. After surgical intervention, where the child is not allowed to eat or drink and is not capable to follow the study-related instructions.
5. Use of any drug that causes nausea, fatigue, or palsy per local prescribing information.
6. Employee of the investigator or study site, with direct involvement in the proposed study or other studies under the direction of that investigator or study site, as well as family members of the employees or the investigator.
7. Any condition for which, in the opinion of the investigator, participation would not be in the best interest of the participant (e.g., compromise the well-being) or that could prevent, limit, or confound the protocol-specified assessments.

Supplementary Table S2. Definitions and outcomes of acceptability and swallowability

Outcome measure	The child swallowed all of the mini-tablets with no chewing[a]	The child chewed or swallowed some of the mini-tablets[b]	The child spat-out the mini-tablets[c]	The child coughed during swallowing[d]	The child refused to take the mini-tablets[e]
**Swallowability**	YES	NO	NO	NO	NO
**Acceptability**	YES	≥80% YES[f]	NO	NO	NO
**Palatability**[g] The child's experience when the mini-tablets were in the mouth	Positive, neutral, or negative	Positive, neutral, or negative	Positive, neutral, or negative	Positive, neutral, or negative	NA

[a]No chewing during deglutition of all mini-tablets with no residuals of the mini-tablets found on oral inspection was interpreted as "accepted and swallowed"; [b]Chewing observed before deglutition and/or the whole or parts of the mini-tablets found on oral inspection was interpreted as "accepted but not swallowed"; [c]No deglutition and the mini-tablets no longer in the child's mouth was interpreted as "not accepted and not swallowed"; [d]Coughing during swallowing of mini-tablets indicated difficulty in swallowing as was interpreted as "not accepted and not swallowed"; [e]The child not allowing the investigator to administer the mini-tablets was interpreted as "not accepted and not swallowed"; [f]Outcome of acceptability assigned as YES if child chewed or swallowed ≥13 and ≥25 mini-tablets in the 16 and 32 mini-tablet administration arms; [g]Palatability was rated based on the following pre-defined criteria: pleasant=positive; no change=neutral; unpleasant=negative.

NA, not applicable.

Supplementary Table S3. Demographic characteristics of study participants by age group.

Characteristic	Group A: 16 mini-tablets with soft food					Group B: 32 mini-tablets with soft food				
**Age group, years**	**2–<3 (n=21)**	**3–<4 (n=19)**	**4–<5 (n=20)**	**5–<6 (n=20)**	**6–<7 (n=20)**	**2–<3 (n=20)**	**3–<4 (n=20)**	**4–<5 (n=20)**	**5–<6 (n=20)**	**6–<7 (n=20)**
**Age, years**										
Mean (SD)	2.0 (0)	3.0 (0)	4.0 (0)	5.0 (0)	6.0 (0)	2.0 (0)	3.0 (0)	4.0 (0)	5.0 (0)	6.0 (0)
Median (range)	2.0 (2, 2)	3.0 (3,3)	4.0 (4, 4)	5.0 (5,5)	6.0 (6, 6)	2.0 (2, 2)	3.0 (3,3)	4.0 (4, 4)	5.0 (5,5)	6.0 (6, 6)
**Sex, n (%)**										
Female	13 (61.9)	11 (57.9)	10 (50.0)	6 (30.0)	8 (40.0)	8 (40.0)	9 (45.0)	9 (45.0)	4 (20.0)	6 (30.0)
Male	8 (38.1)	8 (42.1)	10 (50.0)	14 (70.0)	12 (60.0)	12 (60.0)	11 (55.0)	11 (55.0)	16 (80.0)	14 (70.0)
**Ethnicity, n (%)**										
Hispanic or Latino	1 (4.8)	1 (5.3)	1 (5.0)	1 (5.0)	1 (5.0)	1 (5.0)	0	0	0	1 (5.0)
Non-Hispanic or Latino	20 (95.2)	18 (94.7)	19 (95.0)	19 (95.0)	19 (95.0)	19 (95.0)	20 (100.0)	20 (100.0)	20 (100.0)	19 (95.0)

Characteristic	Group C: 16 mini-tablets with water					Group D: 32 mini-tablets with water				
**Age group, years**	**2–<3 (n=10)**	**3–<4 (n=10)**	**4–<5 (n=10)**	**5–<6 (n=10)**	**6–<7 (n=10)**	**2–<3 (n=10)**	**3–<4 (n=10)**	**4–<5 (n=10)**	**5–<6 (n=10)**	**6–<7 (n=10)**
**Age, years**										
Mean (SD)	2.0 (0)	3.0 (0)	4.0 (0)	5.0 (0)	6.0 (0)	2.0 (0)	3.0 (0)	4.0 (0)	5.0 (0)	6.0 (0)
Median (range)	2.0 (2, 2)	3.0 (3, 3)	4.0 (4, 4)	5.0 (5, 5)	6.0 (6, 6)	2.0 (2, 2)	3.0 (3, 3)	4.0 (4, 4)	5.0 (5, 5)	6.0 (6, 6)
**Sex, n (%)**										
Female	5 (50.0)	4 (40.0)	6 (60.0)	3 (30.0)	2 (20.0)	6 (60.0)	5 (50.0)	4 (40.0)	6 (60.0)	0
Male	5 (50.0)	6 (60.0)	4 (40.0)	7 (70.0)	8 (80.0)	4 (40.0)	5 (50.0)	6 (60.0)	4 (40.0)	10 (100.0)
**Ethnicity, n (%)**										
Hispanic or Latino	1 (10.0)	0	0	0	0	0	0	0	0	0
Non-Hispanic or Latino	9 (90.0)	10 (100.0)	10 (100.0)	10 (100.0)	10 (100.0)	10 (100.0)	10 (100.0)	10 (100.0)	10 (100.0)	10 (100.0)

n, number of evaluable subjects; SD, standard deviation.

Supplementary Table S4. Acceptability, swallowability, and palatability for 16 placebo mini-tablets administered with soft food compared with administration with water by age group.

**Characteristic**	**Group A: 16 mini-tablets with soft food**						**Group C + D: 16 (C) and 32 (D) mini-tablets with water**					
**Age group, years**	**2–<3 (n=21)**	**3–<4 (n=19)**	**4–<5 (n=20)**	**5–<6 (n=20)**	**6–<7 (n=20)**	**Total (n=100)**	**2–<3 (n=20)**	**3–<4 (n=20)**	**4–<5 (n=20)**	**5–<6 (n=20)**	**6–<7 (n=20)**	**Total (n=100)**
**Endpoint, n (%)**												
**Acceptability**[a]	16 (76.2)	16 (84.2)	18 (90.0)	20 (100)	18 (90.0)	88 (88.0)	15 (75.0)	14 (70.0)	15 (75.0)	19 (95.0)	17 (85.0)	80 (80.0)
Difference (95% CI) vs. Group C + D	1.19 (-25.4, 28.0)	14.21 (-13.3, 40.0)	15.00 (-9.8, 39.4)	5.00 (-11.9, 23.9)	5.00 (-18.2, 28.3)	8.00 (-2.3, 18.4)	—	—	—	—	—	—
**Swallowability**[b]	13 (61.9)	8 (42.1)	14 (70.0)	11 (55.0)	7 (35.0)	53 (53.0)	9 (45.0)	8 (40.0)	4 (20.0)	9 (45.0)	12 (60.0)	42 (42.0)

Difference (95% CI) vs. Group C + D	16.90 (-13.7, 44.6)	2.11 (-28.0, 31.9)	50.00 (19.4, 71.9)	10.00 (20.7, 38.9)	-25.00 (-51.8, 6.3)	11.00 (-2.9, 24.5)	—	—	—	—	—	—
**Palatability[c]**	18 (85.7)	17 (89.5)	18 (90.0)	19 (95.0)	18 (90.0)	90 (90.0)	15 (75.0)	14 (70.0)	16 (80.0)	19 (95.0)	18 (90.0)	82 (82.0)
Difference (95% CI) vs. Group C + D	10.71 (-14.7, 35.8)	19.47 (-6.9, 44.1)	10.00 (-14.0, 34.0)	0 (-19.7, 19.7)	0 (-22.3, 22.3)	8.00 (-1.7, 18.0)	—	—	—	—	—	—

[a]Acceptability was defined as swallowing all of the mini-tablets with no chewing, or swallowing ≥80% of the mini-tablets with or without chewing; [b]Swallowability was defined as swallowing all of the mini-tablets without chewing and no residuals of the mini-tablets found in oral inspection; [c]For assessment of palatability, the immediate reactions of the child after administration of the mini-tablets were recorded as positive (pleasant), neutral (no change), or negative (unpleasant) according to the pre-defined criteria. A positive or neutral reaction qualified as palatable.

— not relevant; CI, confidence interval; n, number of evaluable subjects.

Supplementary Table S5. Acceptability, swallowability, and palatability for 32 placebo mini-tablets administered with soft food compared with administration with water by age group.

Characteristic	Group B: 32 mini-tablets with soft-food						Group C + D: 16 (C) and 32 (D) mini-tablets with water					
**Age group, years**	**2–<3 (n=20)**	**3–<4 (n=20)**	**4–<5 (n=20)**	**5–<6 (n=20)**	**6–<7 (n=20)**	**Total (n=100)**	**2–<3 (n=20)**	**3–<4 (n=20)**	**4–<5 (n=20)**	**5–<6 (n=20)**	**6–<7 (n=20)**	**Total (n=100)**
**Endpoint, n (%)**												
**Acceptability[a]**	13 (65.0)	17 (85.0)	18 (90.0)	17 (85.0)	19 (95.0)	84 (84.0)	15 (75.0)	14 (70.0)	15 (75.0)	19 (95.0)	17 (85.0)	80 (80.0)
Difference (95% CI) vs Group C + D	-10.00 (-37.4, 18.8)	15.00 (-11.8, 40.5)	15.00 (-9.8, 39.4)	-10.00 (-32.4, 11.3)	10.00 (-11.3, 32.4)	4.00 (-6.8, 14.8)	—	—	—	—	—	—
**Swallowability[b]**	9 (45.0)	12 (60.0)	8 (40.0)	7 (35.0)	10 (50.0)	46 (46.0)	9 (45.0)	8 (40.0)	4 (20.0)	9 (45.0)	12 (60.0)	42 (42.0)
Difference (95% CI) vs Group C + D	0 (-29.8, 29.8)	20.00 (-11.2, 47.6)	20.00 (-8.9, 46.2)	-10.00 (-38.5, 20.2)	-10.00 (-38.8, 20.6)	4.00 (-9.7, 17.6)	—	—	—	—	—	—
**Palatability[c]**	13 (65.0)	18 (90.0)	18 (90.0)	16 (80.0)	19 (95.0)	84 (84.0)	15 (75.0)	14 (70.0)	16 (80.0)	19 (95.0)	18 (90.0)	82 (82.0)
Difference (95% CI) vs Group C + D	-10.00 (-37.4, 18.8)	20.00 (-5.6, 44.5)	10.00 (-14.0, 34.0)	-15.00 (-38.0, 7.1)	5.00 (-15.5, 26.4)	2.00 (-8.6, 12.6)	—	—	—	—	—	—

[a]Acceptability was defined as swallowing all of the minitablets with no chewing, or swallowing ≥80% of the mini-tablets with or without chewing; [b]Swallowability was defined as swallowing all of the mini-tablets without chewing and no residuals of the mini-tablets found in oral inspection; [c]For assessment of palatability, the immediate reactions of the child after administration of the mini-tablets were recorded as positive (pleasant), neutral (no change), or negative (unpleasant) according to the pre-defined criteria. A positive or neutral reaction qualified as palatable.

—, not relevant; CI, confidence interval; n, number of evaluable subjects.
